# Molecular surveillance of arboviruses in Nigeria

**DOI:** 10.1186/s12879-023-08526-z

**Published:** 2023-08-18

**Authors:** Joseph Ojonugwa Shaibu, Kabiru Olusegun Akinyemi, Oshilonyah Henry Uzor, Rosemary Ajuma Audu, Akeeb Bola Oriowo Oyefolu

**Affiliations:** 1 Department of Microbiology, Faculty of Sciences, Lagos State University, Ojo, Lagos State Nigeria; 2 Centre for Human Virology and Genomics, Nigerian Institute of Medical Research, Yaba, Lagos State Nigeria; 3 Department of Microbiology, University of Delta, Agbor, Delta State Nigeria

**Keywords:** Surveillance, Yellow fever, Virus, Arbovirus, Molecular, Sequencing

## Abstract

Arboviral infections are fast becoming a global public health concern as a result of its high fatality rate and sporadic spread. From the outbreak of Zika virus in the Americas, the endemicity of Yellow fever in West Africa and South America, outbreaks of West Nile virus in South Africa to the year-round and national risk of Dengue fever in Mainland China and India. The war against emerging and re-emerging viral infection could probably lead to the next pandemic. To be above the pending possible arboviral pandemic, consistent surveillance of these pathogens is necessary in every society. This study was aimed at conducting a surveillance for Yellow fever virus, Zika virus, Chikungunya virus, Dengue virus and Rift Valley fever virus in four states in Nigeria using molecular techniques. A cross-sectional study involving 1600 blood samples collected from febrile patients in Lagos, Kwara, Ondo and Delta States between 2018 and 2021 was conducted using Real time polymerase chain reaction for detection of the pathogens. Extraction and purification of viral RNA were done using Qiagen Viral RNA Mini Kit. Samples were analyzed using One Step PrimeScript III RT-PCR mix (Takara Bio) alongside optimized primers and probes designed in-house. Positive samples were sequenced on MinION platform (Nanopore technologies). Bioinformatic and phylogenetic analysis were performed with DNASTAR Lasergene 17.3. All the RNA extracted from samples collected from the four states were negative for ZIKV RNA, RVFV RNA, CHIKV RNA and DENV RNA. However, twelve of the samples (2%) tested positive for YFV RNA. Three full genomes of sizes 10,751 bp, 10,500 bp and 10,715 bp were generated and deposited in GenBank with accession numbers: ON323052, ON323053 and ON323054 respectively. Phylogenetic analysis shows clustering within lineage 3 of West African genotype. This result shows an active spread of Yellow fever in Delta State, Nigeria. However, there is no emergence of a new genotype There is a need for an intense surveillance of Yellow fever virus in Nigeria to avert a major outbreak.

**Supplementary Information:**

The online version contains supplementary material available at 10.1186/s12879-023-08526-z.

## Introduction

Arboviruses are arthropod or insect-borne viruses classified under four major families: Bunyaviridae, Togaviridae, Flaviviridae and Reoviridae. Examples are; Yellow fever virus (YFV), Zika Virus (ZIKV) and Dengue virus (DENV) {the three belong to the family Flaviviridae}; Chikungunya virus (CHIKV) {belongs to Togaviridae family}; Rift valley fever virus (RVFV) and Crimean Congo Haemorrhagic Fever Virus (CCHFV) {both belong to Bunyaviridae family}. The incubation period varies from virus to virus, but is usually limited between 2 and 15 days for arboviruses [1]. The most common clinical features of infection are fever, headache, and malaise, but encephalitis and viral hemorrhagic fever may also occur [2].

The arboviruses spread mainly through insect bites with the most common been mosquito. It is reported that the viruses can also spread through: blood transfusion, organ transplant, sexual contact, pregnancy and childbirth from mother to child [3–6]. On a global scope, dengue virus may be the most challenging, infecting 100 to 390 million people and leading to 12,500 deaths per year [7]. Zika virus has been implicated in complications such as Guillain-Barre syndrome and other neurological disorders in adults, microcephaly, severe brain malformations, and other birth defects [8]. In November 2020, NCDC reported an outbreak of Yellow Fever in Delta State. Out of 48 suspected cases, 30 deaths (CFR 62.5%) were recorded [9].

The emergence and re-emergence of these infections have been hinged of factors such as urbanization and rapid population growth which results in human crowding, higher waste generation, and provision of conducive environment for the thriving of vectors of these viruses. In addition, human-human transmission has been aided by rapid rise in movement of people from one country to another. In most rural areas in Nigeria, screening for infections or disease manifestation with febrile feelings are limited to either malaria and/or typhoid. In cases where the results come out negative for these pathogens, there are no further screenings for possible viral infections. The patients whose immune systems were able to fight the infection survive and those who could not die. This kind of death has been tagged as “mysterious” death superstitiously.

In Nigeria and other low-income countries, there is paucity of information on molecular characterization of these viruses as a result of limited researches. Most often, because of fund limitations, studies are restricted only to serological studies to investigate the level of exposure of people to these viruses. However, serological techniques are limited with respect to detection of active infection, tracking of strains in circulation, identifying mutations and their possible effect on vaccines, drugs and diagnostics. Hence, this study was aimed at conducting surveillance for Zika, Yellow, Rift Valley, Dengue and Chikungunya viruses in Nigeria using molecular methods.

## Materials and methods

### Ethical consideration

The ethical approval (IRB-17-049) for this studied was obtained from Institutional Review Board of Nigerian Institute of Medical Research (NIMR), Yaba. Informed consents were sought from the participants by them filling the informed consent form and understood what the project is all about. Personal details of the participants were delinked from this study. At the site of sample collection, the ethical approval obtained from NIMR -IRB was presented to the authority in-charge of the hospital for approval to collect samples. The information gathered from participants were kept confidential. Their names were delinked from the outcome of the research on their samples.

### Study design/area

This is a cross sectional study aimed at molecular detection and characterization of some arboviruses among febrile patients. The samples were collected from hospitals in Delta State, Lagos State, Ondo State and Kwara State. Lagos and Ondo State are in the South Western part of Nigeria, Kwara State is in the North Central and Delta State in the South Southern part. The choice of these states was on the previous records of arboviral infections reported by researchers and Nigerian Centre for Disease prevention and Control (NCDC) and a chance of higher effect if there is an outbreak of any of these viruses. In Delta state, the samples were collected from Ika North-East LGA, in Lagos, from Alimosho, Shomolu, Isolo, Surulere and Gbagada general hospitals, in Ondo, from Federal medical centre, Owo; in Kwara, from Ilorin West LGA. These hospitals are where most of referrals in the states are taken to. People from both urban and rural parts of the states visit the hospitals where samples were collected, however, majority of the participants are from a semi-urban settlement. Participants’ demographic information (age and gender) were also collected.

### Sample collection/processing

A total of 1600 blood samples were collected from febrile patients who attended the health centres under sampling for treatment in the period between 2018 and 2021 using a simple convenience sampling method. The samples were collected in 10 millilitres EDTA vacutainer tubes, and transported to the laboratories in the various states for separation. Plasma were then transported in triple package to Centre for Human Virology and Genomics (ISO 15,189 accredited), Nigerian Institute of Medical Research, Yaba, Lagos state, Nigeria for storage at -80^o^C and further processing.

#### RNA EXTRACTION

The viral RNA were extracted using Viral RNA + DNA preparatory kit (Jena Biosciences, Germany) in accordance to the manufacturers’ instructions.

### RT-qPCR amplification

One-step reverse transcriptase (RT) real-time (qPCR) was carried out to detect the pathogens in a single run for each of the viruses using optimized primers and probes (Table 1). A total volume of 25ul containing: 12.5 ul One-Step PrimeScript mix (Takara bio, France), 0.8ul each for forward and reverse primers, 0.6ul for probe, 5.3ul for Rnase free water and 5ul of RNA template was ran on Quant Studio 5 (Applied Biosystems) as follows: 52 °C for 10 min, 95 °C for 10 s, then 45 cycles of 95^o^C for 5s and 56^o^C for 45s.

**Table 1:** Primers and probes for the detection of selected arboviruses

S/N	Pathogen	Primers and probes		Reference
1	Zika	Forward	AARTACACATACCARAACAAAGTG GT	[10]
		Reverse	TCCRCTCCCYCTYTGGTCTTG	
		Probe	FAM - CTYAGACCAGCTGAAR	
2	Yellow fever	Forward	AAGATCCTGTGAAGCTTGCAT	In-house assay
		Reverse	CCTCGTTTTCCTCAAGAATGG	
		Probe	FAM - GAAGTGTGGCCTAAATTCAGTTGACTCCCT	
3	Dengue virus	Forward	AAACCGCGTGTCGACTGTGC	In-house assay
		Reverse	TAGGAAACGAAGGAATGCCACC	
		Probe	FAM-5’CACTTGGAATGCTGCAGGGACGAGGACC	
4	Rift valley fever virus	Forward	CGGACTTGGAGACTTTGCATCA	[11]
		Reverse	TTCTTTCAGATTGGGGAACCTTGT	
		Probe	FAM - ACGTTGCACCTCCACCAGCGAAGC	
5	Chikungunya virus	Forward	TCACTCCCTGTTGGACTTGATAGA	[12]
		Reverse	TTGACGAACAGAGTTAGGAACATACC	
		Probe	FAM – 5’ AGGTACGCGCTTCAAGTTCGGCG	

#### cDNA SYNTHESIS AND MULTIPLEX PCR

Synthesis of highly structured and long cDNA fragments was performed using SCRIPT cDNA Synthesis Kit (Jena Bioscience, GmbH, Germany) according to the manufacturer’s instructions. To enhance the amplicon yield and to concentrate amplifications along the multiple primers, a multiplex PCR was conducted using the pairs of in-house designed primers in (Table 2) (designed from conserved regions using Oligo Primer Analysis Software v.7). In the PCR process, a total volume of 20ul of reaction mix was prepared with; 3.7ul of nuclease-free water, 0.05ul of primers (Table 2), 6.25ul of Q5 HS PCR mix and 10ul of cDNA each for pool 1 and pool 2 using the cycling condition of 98^o^C for 30 s, 35 cycles of 98^o^C for 15 s and 65^o^C for 5 min, and 4^o^C for infinity on MiniAmp plus thermal cycler by Thermofischer scientific.

**Table 2:** Comparison of percentage identity of consensus sequences with YFV whole genome sequences of Nigerian origin mined from NCBI

S/N	Accession numbers/Location	ON323052 (%)	ON323053 (%)	ON323054 (%)
1	MN211302/Edo	97.66	97.19	96.63
2	MN211306/Edo	97.66	97.18	96.63
3	MN211311/Edo	97.67	97.2	96.65
4	MK457701/Edo	97.66	97.2	96.64
5	MN958078/Edo	97.63	97.19	96.61
6	MN211307/Edo	97.68	96.29	96.17
7	MN211303/Edo	97.12	97.69	96.68
8	MN211304/Edo	97.7	97.23	96.68
9	MN211308/Edo	97.61	97.18	96.58
10	MN211301/Edo	97.7	97.23	96.68
11	MN211310/Edo	97.69	97.24	96.67
12	KU978763/Ogbomosho	91.4	91.02	91.2

### MinION sequencing

Sequencing run was carried out on MinION (Nanopore Technology) using a procedure that was developed by Quick et al., 2017 to sequence whole genome of Zika virus during 2016 outbreak. Using the pipeline, 35 pairs of primers (Table 3) were generated to cover the whole length of YFV, the primers were pooled with the odd numbers in one tube and the even number in the other. The pooled primers were used in multiplex PCR as stated earlier to generate amplicons with respect to each pair of the primer used in the pools. The amplicons from both pools were then pooled together to serve as the DNA template for the library preparation for sequencing. The library preparation was carried out using SQK-RBK110-96 kit with a flow cell (FLO-MIN106) following kit manufacturer’s instructions which entails four basic processes: multiplex PCR, barcoding of samples, pooling of samples and clean up, and priming and loading of flow cell.

**Table 3:** List of primers used for sequencing

S/N	Primer Code	Sequence	Tm	S/N	Primer Code	Sequence	Tm
1	YFVM_1F	TCCTGTGTGCTAATTGAGGTGC	61.06	36	YFVM_18R	GCTCTGATGGATGGAAGGAACC	60.93
2	YFVM_1R	GGCGTTTCCTTGAGGACAATCC	61.95	37	YFVM_19F	TTCAAACGGACATACCCAGCG	61.04
3	YFVM_2F	GGAAAATGCTGGACCCAAGACA	61.26	38	YFVM_19R	AGTCTCCATCTCTGTTGGGGTT	60.95
4	YFVM_2R	CTGCTGAGTCACACTTGCCATA	60.79	39	YFVM_20F	AGGAAGGTGGCAATAAAGGGG	60.1
5	YFVM_3F	ATCTCAGTCCAAGAGAGGAGCC	61.14	40	YFVM_20R	AGATCTCATGTTCCTCAGGGCC	61.74
6	YFVM_3R	CTGAAACCCAGGTTCCTCCATG	61.06	41	YFVM_21F	AATTGTGACCTGCCCGTCTG	60.62
7	YFVM_4F	TGTTGGTCCGGCTTACTCAG	59.7	42	YFVM_21R	GACTATTGTCATTGCCTCAGGCA	61.19
8	YFVM_4R	ACAAGCTCATGGATTTGGCACA	61.27	43	YFVM_22F	CCATCAGTGTGTTCCTCCACTC	60.79
9	YFVM_5F	GATAGAGGCTGGGGTAATGGCT	61.55	44	YFVM_22R	GTTTTCTCCAGCATGCCTAGCT	61.13
10	YFVM_5R	ATGATGCATCTCTCTCCACACC	60.08	45	YFVM_23F	ATCCAAGACAACCAAGTGGCAT	60.68
11	YFVM_6F	AGCTGGATAGTGGACAGACAGT	60.74	46	YFVM_23R	ACAGAGCAAAGGCATCACTGTT	60.93
12	YFVM_6R	CTATCACTGGGATCTTGCAGGG	60.41	47	YFVM_24F	CCTTTCTTTCATGGACAAGGGGA	60.76
13	YFVM_7F	CAAGAACCCAACTGACACTGGT	60.87	48	YFVM_24R	AGAAGGCTGGTGTTTCCCTCTA	60.95
14	YFVM_7R	GCAGAGCCAAACACCGTATGAA	61.63	49	YFVM_25F	ATGTGCAGAACGCCCTTTTCA	61.19
15	YFVM_8F	AGACGCCGCCTGGGATTTTA	61.92	50	YFVM_25R	CACGCTCATGGAACCACCTTAA	61.05
16	YFVM_8R	ACTTCCCTTCTTCAAAAGAGGCT	60.12	51	YFVM_26F	CTGACATTGTGGAGGTGGATCG	61.18
17	YFVM_9F	GGGACTCTGATGATTGGCTAAACA	60.95	52	YFVM_26R	AGAACTCTCATGGTCCTCTCCC	60.81
18	YFVM_9R	TCCCGTCCCAAACTCCTCTATC	61.14	53	YFVM_27F	TTGGAACCAGTGAAATGCGACA	61.19
19	YFVM_10F	TGGAAGCTTTATCATAGATGGAAAGTCT	60.93	54	YFVM_27R	ATCTGTCTCAACACTGCGTGTC	61.04
20	YFVM_10R	GTTCGTCTGAACCTTGTACCCA	60.4	55	YFVM_28F	GAATGAGGCGTCCAACTGGAAA	61.31
21	YFVM_11F	AGAGTGAAATGTTCATGCCGAGA	60.56	56	YFVM_28R	TTCCTAGTTCCTGCTGGTGGAT	61.02
22	YFVM_11R	GCTATCATCATGCTCACCAAACC	60.25	57	YFVM_29F	TGACACAACCCCTTTTGGACAG	61.13
23	YFVM_12F	CCAAGGAAAACACATGAAAGCCA	60.44	58	YFVM_29R	ATGTACCATATGGCACGGCTTC	60.99
24	YFVM_12R	CAATCTCCACCATGGCTGCT	60.41	59	YFVM_30F	GAGGTGTCGGACTTGTGTGTAC	61.04
25	YFVM_13F	TTTTCAATCAGACCAGGGCTGC	61.58	60	YFVM_30R	GGCTGGTCTCAACACTTTCACT	60.93
26	YFVM_13R	GAAATGCACACAGGCCCAAAAA	60.93	61	YFVM_31F	TCCTGAACTACATGAGCCCACA	61.28
27	YFVM_14F	CCTCCATGCAGAAGACCATACC	60.67	62	YFVM_31R	TCTAACCTTGGACATGGCGTTG	61.05
28	YFVM_14R	GTCCCATGGCACTTTCTCTTCA	60.74	63	YFVM_32F	GGTGAGTGGAGACGATTGTGTT	60.73
29	YFVM_15F	ATGGGAAGAGGAGGCAGAGATC	61.21	64	YFVM_32R	ACCATGTTGTGCGTCCTTGTG	61.78
30	YFVM_15R	TGCCATTTCTGACAAGGAAGGC	61.58	65	YFVM_33F	AGGGACATGAGACTGCTGTCAT	61.34
31	YFVM_16F	AGTCAACCTTCTTGGGGGCTT	61.72	66	YFVM_33R	GTTTCAGATAAGCTCACCCGGT	60.54
32	YFVM_16R	GACACGAAGGAGTTGTCACCAA	60.92	67	YFVM_34F	GGACAGGAGAAATACACTGACTACC	60.78
33	YFVM_17F	AGTGGCACTTCAGGATCTCCTA	60.48	68	YFVM_34R	AGGCTCCGTTCTTTTTACTCTGG	60.81
34	YFVM_17R	GGCATGACACATGGCATCAATG	60.98	69	YFVM_35F	ACAACCGGGATACAAACCACG	60.98
35	YFVM_18F	GAAAGAGGCTTTTCACGGCTTG	60.78	70	YFVM_35R	GGTCTTTCCCTGGCGTCAATA	60.17

### Sequence assembly

The fastQ files were copied and analyzed using DNASTAR Lasergene v.17.3 software. The assemblage was carried out relative to the reference strain. Base calling for variant analysis was carried out at Qcall = 30% and minimum depth coverage of 5.

### Phylogenetic analysis

Forty-one (41) sequences of YFV which cut across the already identified genotypes across Africa were mined from NCBI GenBank database and aligned alongside the consensus sequences (ON323052, ON323053 and ON323054) assembled from this study, using MAFFT vs7.1 Alignment software. Phylogenetic tree was constructed using same software by applying Neighbor-joining method [13] and Jukes-cantor model with Bootstrap resampling at 1000. The tree was viewed on archaeopteryx.js software and downloaded on Newick.

## Results

Out of the 1600 samples analyzed, 450(28%) were collected from Lagos State, 400(25%) from Delta State, 400(25%) from Kwara State, and 350(22%) from Ondo State. Averagely, 480(30%) were males while 1120(70%) were females. RT-qPCR analysis of all the RNA extracted from samples collected from the four states were negative for ZIKV RNA, RVFV RNA, CHIKV RNA and DENV RNA. However, twelve of the samples (0.75%) (when compared with the total number of samples screened across the four states) tested positive for YFV RNA (Ct values: 32–38) while others where negative for YFV RNA. The twelve positive samples were from Delta state. Of the positive samples, 10(83.3%) are males while 2(16.7%) are females within the age range of 20–29 years. Clinical symptoms observed among cases that tested positive for YFV RNA were: jaundice (58.3%), body pain (75%), fever above 38^o^C (100%), vomiting (75%), bleeding from nose, gums and eyes (25%). However, majority of the participants from Delta state had general body pains, fever, headache and vomiting but tested negative for all the viruses. None of the samples tested positive for two or more pathogens analyzed though they all share common vector.

### Sequencing

Sequencing was performed on three samples out of the twelve positives. These three samples had the lowest ct values (< 30) and also the ones that showed bands on gel after conventional PCR. In addition, two of the participants from which the samples were collected had severe manifestations of bleeding from the orifices in addition to the other symptoms which were common to the rest. (Table 2). Sequencing was run for 19 h and 14 min, one million and fifty thousand (1.05 M) reads were generated with a total passed bases of 136.44 Mb out of an estimated 363.35 Mb (37.5%). The quality score for the base calling was around the 75% quartile.

### Sequence assembly

The assembled sequences were successfully deposited in NCBI public database and have been assigned the following accession numbers: ON323052, ON323053 and ON323054 for sample codes YFV-1, YFV-2 and YFV-3 with sizes 10,751 bp, 10,500 and 10,715 bp respectively. The percentage identity of ON323052, ON323053 and ON323054 relative to NC_002031.1 are 98.33%, 98.54% and 97.98% respectively. Comparison of percentage identities of the consensus sequences with other full genome sequences of Nigerian origin on NCBI is summarized on Table 2.

### Phylogenetic analysis

**Fig. 1:**
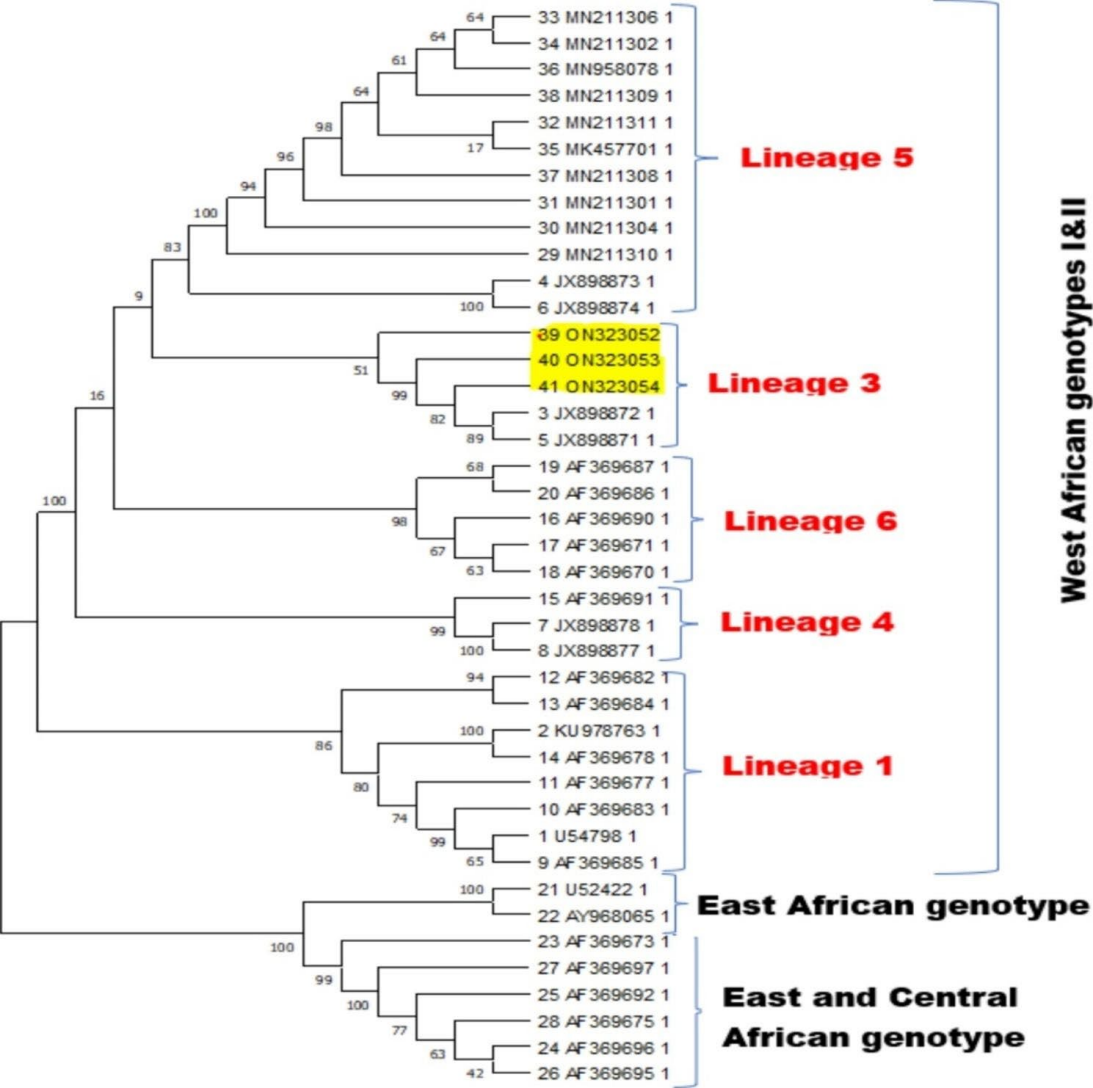
Phylogenetic analysis of the consensus sequences (ON323052, ON323053, ON323054) in relation to other sequences across West Africa

**Fig. 2:**
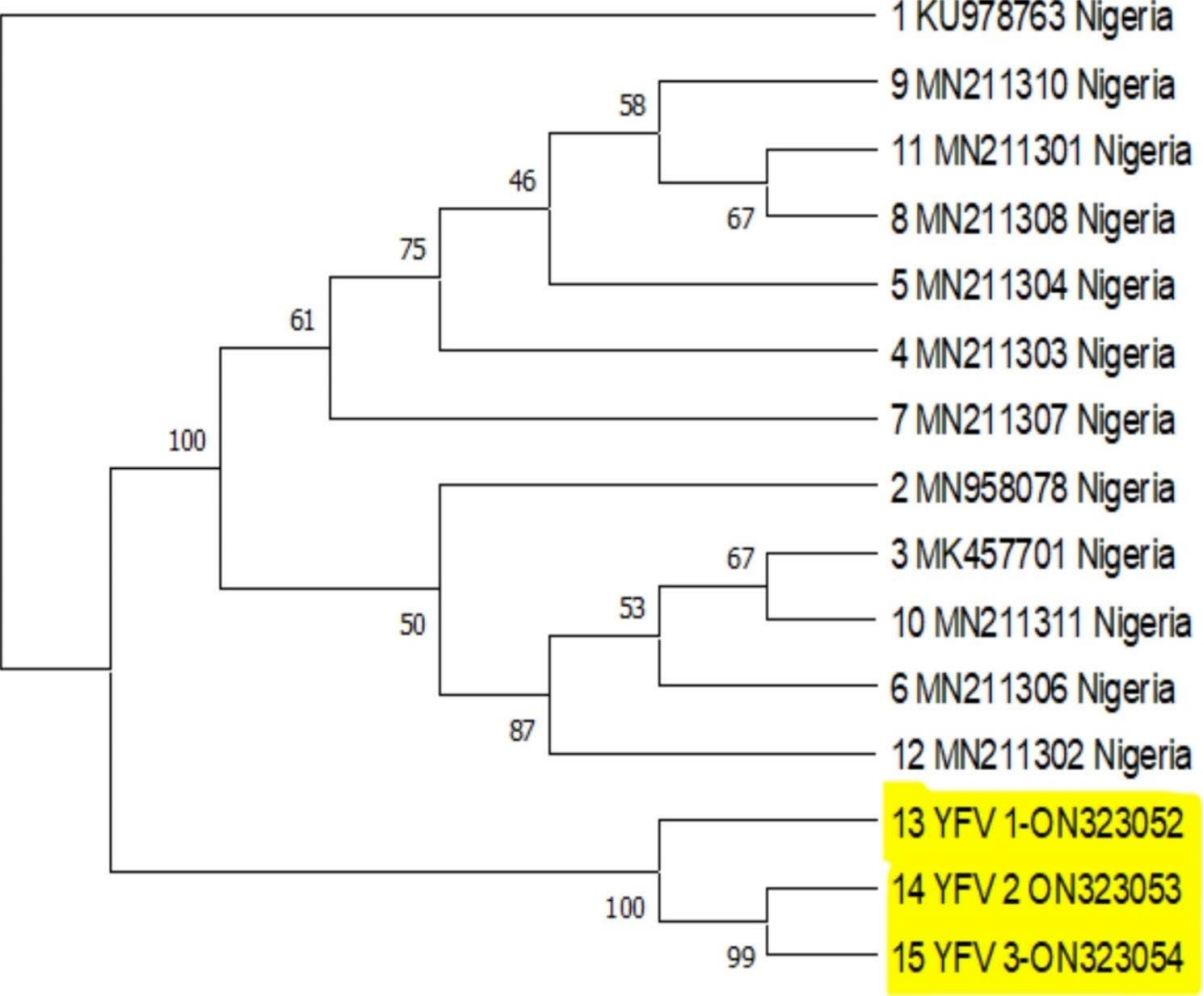
Phylogenetic analysis of the consensus sequences (ON323052, ON323053, ON323054) in relation to other whole genome sequences across Nigeria mined from NCBI. It shows a relatively different strain from the previous ones

Analysis of the consensus strains alongside other sequences generated across West Africa showed a clustering around Lineage 3 of the West African genotype. They revealed close relationship with other strains from Senegal (JX898871, JX898872) (Fig. 1). Figure 2, phylogeny analysis indicated that the three new consensus sequences are different strains for the previously identified ones. It showed that ON323053 and ON323054 are more closely related relative to ON323052.

## Discussion

The threat from infections of arboviral origin are fast becoming a global concern. Most infections that were at a time localized are beginning to spread out to several nations of the world and hence becoming global threat to World Health Organization and other national health organizations. In the list of WHO priority diseases, arboviral infections are of majority. In Nigeria, Nigeria Centre for Disease Control (NCDC) has highlighted mosquito-borne infections arising from viruses such as Yellow fever, Dengue, Chikungunya, Rift Valley fever and other viral haemorrhagic fever viruses as emerging diseases of concern and under surveillance. Of the infections from these viruses, yellow fever, a disease caused by yellow fever virus has been a consistent concern with sporadic outbreak yearly. Serological evidence of these viruses has been recorded in previous studies conducted in some states across the country with seroprevalences of 16.4–32.4% [14–16] for CHIKV; 21.5% [17] for RVFV; 11.1–50% [18–20] for YFV and 2-55.6% [18, 20–22]. However, there is limited molecular information on these virus neccesiating this molecular surveillance study to investigate the real time true burden of these infections (which most times have been misdiagnosed) within Nigeria. The PCR results from the screening of the viruses in this study signifies that as at the period the samples were collected, there were no active spread of Zika, Chikungunya, Dengue and Rift valley fever viruses in the four states sampled. Although the individuals whose samples were analyzed had fever, it could be deduced that the fever did not arise from these viral infections. Also knowing that the viruses are RNA viruses, mode of sample storage before and during transportation is important to maintaining the integrity of the RNA. Furthermore, it could imply that the participants were already at the recovery phase of the infection and as such; viral loads below the limit of detection of the assays.

However, 12(0.75%) of the total number of samples collected from the four states tested positive for YFV. This samples were all from Delta state implying an active prevalence of 3% of the population from the state. This signified an active spread of yellow fever in Delta state which agreed with the fact that Delta state has being endemic for yellow fever in the past few years as reported by NCDC and WHO [9]. Environmental conditions of the residential areas could play a role in nurturing vectors; usually, people living in rural areas where there are bushes, stagnant bodies of water, untidy dark spot at corners and limited access to the use of insecticides and mosquito nets are more exposed to mosquito bite and consequently, a higher chance of coming down with fever. Although fever as a symptom is not only limited to mosquito bites, however, in Africa, most fever cases could be attributable to mosquito bite. Narrowing down to Delta state, it could be deduced that regular exposure to mosquito especially of the *Aedes sp* which is the vector of YFV, is a predisposing factor to being infected with the virus which could manifest in fever. Apart from fever, other symptoms such as jaundice (58.3%), general body pains (75%), bleeding from nose, gum and eyes (25%), headache (75%) and vomiting (75%) reported among the participants are clear signs of probable active spread of YFV in these communities. The negative samples recorded within the same communities pointed to a possibility that at the time of sample collection, some of the patients have shed the virus and the viral was no longer at the level to be detected.

This study also reported a higher prevalence of active YFV infection among males (83.3%) than females (16.7%) in Delta state. This could be as a result of certain habits of males such as farming and hunting in bushes, staying out late in the dark and exposure of their bodies while sleeping [16]. These habits predispose them to regular mosquito bites and human-human transmission of infections.

Furthermore, the clustering of the sequences around lineage 3 (around strains from Senegal (JX898872 & JX898871) is contrary to previous reports of the circulation of lineage 1 and lineage 5 in Nigeria [23]. This could be a possible hint of the route or source of infection and possible gradual emergence of a sub-lineage.

Despite the clustering around a lineage that is not usual in previous reports in Nigeria, there is no sign of emergence of a new genotype of YFV circulating in Delta state from this study.

## Conclusion

This study has revealed that there is currently no active circulation of DENV, ZIKV, CHIKV and RVFV in the four states where the study was conducted. However, it reported an active spread of YFV of Lineage 3 in Delta state which is a deviation from the regular lineages previously identified in Nigeria. There is a possibility of a future sub-lineage emerging.

### Recommendation

In order to effectively prevent, control or manage disease outbreak, routine surveillance is required to investigate levels of exposure and active infection with the population. There is a need for further studies to track the origin of the lineage 3 of the virus in circulation. On the national level, a nationwide surveillance of these viral pathogens across Nigeria to understand the true burden is necessary for effective management of febrile cases.

### Electronic supplementary material

Below is the link to the electronic supplementary material.

Supplementary Material 1

Supplementary Material 2

Supplementary Material 3

## Data Availability

Sequences data generated from this study are available in NCBI GenBank with accession numbers: ON323052, ON323053 and ON323054. The sequences are attached as supplementary documents.

## Abbreviations

DENV: Dengue virus
CHIKV: Chikungunya virus
RVFV: Rift valley fever virus
YFV: Yellow fever virus
ZIKV: Zika virus
NCDC: Nigerian Centre for Disease prevention and control
WHO: World health organization
